# Bioelectronic modulation of the thymic “genetic mirror”: 448 kHz radiofrequency stimulation as a novel strategy for immune tolerance induction in type 1 diabetes

**DOI:** 10.3389/fimmu.2026.1851942

**Published:** 2026-06-15

**Authors:** Natasha Yaneva

**Affiliations:** 1 Clinic for Paediatric endocrinology, diabetes and metabolic diseases, University Children’s Hospital “Prof. Ivan Mitev”, Sofia, Bulgaria; 2 Department of Paediatrics, Medical Faculty, Medical University of Sofia, Sofia, Bulgaria

**Keywords:** 448 kHz radiofrequency, AIRE protein, bioelectronic medicine, capacitive-resistive electric transfer (CRET), central immune tolerance, helios transcription factor, regulatory T-cells (Tregs), type 1 diabetes mellitus

## Abstract

**Background:**

Type 1 Diabetes Mellitus (T1DM) arises from a fundamental breakdown of central immune tolerance within the thymus. Medullary thymic epithelial cells (mTECs) express the Autoimmune Regulator (AIRE) protein, which drives the promiscuous transcription of peripheral tissue-restricted antigens — including insulin — thereby creating a “genetic mirror” essential for the clonal deletion of autoreactive T-cells. Genetic susceptibility loci that reduce intrathymic insulin expression allow high-affinity insulin-reactive T-cells to escape negative selection and enter the periphery, where they ultimately orchestrate the progressive destruction of pancreatic β-cells. Despite decades of research, no therapeutic strategy has yet succeeded in restoring this upstream tolerogenic defect at its source.

**Hypothesis:**

I hypothesise that the thymic microenvironment can be bioelectronically reprogrammed to re-establish central immune tolerance in T1DM. Specifically, I propose that non-invasive application of 448 kHz radiofrequency (RF) fields, delivered via a wearable smart patch using subthermal Capacitive-Resistive Electric Transfer (CRET), can selectively upregulate *AIRE*-dependent insulin expression in mTECs, thereby restoring the intrathymic “immunological shadow” of the pancreatic β-cell.

**Proposed mechanism:**

The 448 kHz RF field is hypothesised to interact with L-type voltage-gated calcium channels (Cav1.2/Cav1.3) on mTEC membranes, inducing a controlled, subthermal Ca²^+^ influx. This calcium signal is proposed to activate two parallel intracellular cascades: the MAPK/ERK pathway and the calcineurin/NFAT–NF-κB axis, both of which converge on transcriptional upregulation of *AIRE* and, consequently, enhanced intrathymic insulin (*Ins2*) expression. Strengthened negative selection and the preferential generation of epigenetically stable Helios^+^Foxp3^+^ thymus-derived regulatory T-cells (tTregs) are predicted as downstream immunological outcomes. The hypothesis is testable and falsifiable through a structured programme of preclinical experiments in the Non-Obese Diabetic (NOD) mouse model, incorporating pharmacological pathway dissection, live-cell calcium imaging and longitudinal immune phenotyping.

**Significance:**

This approach represents a conceptual shift from peripheral immunosuppression or symptomatic glycaemic management towards upstream, disease-modifying bioelectronic intervention. By targeting the embryologically conserved thymic–pancreatic axis, the 448 kHz CRET strategy offers a non-invasive, paediatric-compatible platform to arrest the autoimmune cascade at its immunological origin, with potential implications for the prevention and early interception of T1DM.

## 1 Introduction: the thymic-pancreatic axis

The developmental synergy between the thymus and the pancreas stems from their shared endodermal origin within the primitive foregut (1, 2). This common embryonic lineage enables the thymus to operate as a specialised “educational hub” where central tolerance is meticulously shaped. Within the thymic medulla, the expression of peripheral tissue-restricted antigens (TRAs) provides a molecular “shadow” of the body’s self-components (3).

In the context of Type 1 Diabetes (T1DM), a disease whose global prevalence and incidence continue to increase at a substantial rate (4), this educational process is fundamentally compromised. Evidence suggests that the degree of central tolerance is directly tied to the levels of intrathymic insulin, which are often modulated by specific genetic susceptibility loci (5, 6). Insufficient insulin expression within this niche allows high-affinity autoreactive T-cells to bypass thymic checkpoints and escape into the periphery (7). Consequently, restoring immunological balance by enhancing ectopic thymic insulin expression and presentation represents a scientifically grounded therapeutic strategy.

## 2 The hypothesis: RF-induced immune tolerance

My hypothesis posits that the thymus can be bioelectrically modulated to recalibrate immune tolerance (8). At the core of this approach is the application of 448 kHz radiofrequency (RF), a clinically validated frequency used in Capacitive-Resistive Electric Transfer (CRET) therapy (9). I hypothesise that this specific frequency can selectively stimulate medullary thymic epithelial cells to enhance thymic insulin expression and presentation, thereby restoring the central tolerance mechanisms that are compromised in T1DM.

### 2.1 Proposed cellular mechanism

The proposed mechanism begins with the interaction of the 448 kHz RF field with voltage-gated calcium channels (VGCCs) on the mTEC surface. The subthermal energy delivered via CRET is hypothesised to induce localised, controlled Ca²^+^ influx without causing thermal damage to surrounding thymic tissue.

Among the voltage-gated calcium channel (VGCC) family, L-type channels — specifically Cav1.2 and Cav1.3 — represent the most plausible molecular candidates for mediating the effects of 448 kHz CRET stimulation on mTECs. L-type calcium channels are widely expressed in epithelial and immune cell populations and have been shown to be sensitive to electromagnetic stimulation within the radiofrequency range. Their voltage-dependent gating properties and relatively slow inactivation kinetics make them well-suited to respond to the pulsed sinusoidal waveform proposed in this intervention.

Experimental validation of L-type VGCC involvement will require a multi-pronged approach. Pharmacological inhibition using selective L-type blockers — such as Nifedipine or Verapamil — can be performed during CRET stimulation in cultured mTECs or thymic organoids. If L-type channels are indeed the primary transducers, blockade should abolish or significantly attenuate downstream signalling responses (e.g., MAPK/ERK activation, *AIRE* upregulation). Live-cell Ca²^+^ imaging using fluorescent indicators (e.g., Fluo-4, GCaMP6) will allow direct visualisation of calcium transients in real time, confirming that CRET exposure induces localised Ca²^+^ influx in mTECs. Patch-clamp electrophysiology can then provide definitive evidence that CRET stimulation modulates Cav1.2 and Cav1.3 activity at the biophysical level. Together, these complementary approaches will establish whether L-type VGCCs serve as the primary molecular gateway for bioelectronic modulation of thymic tolerance.

#### 2.1.1 The MAPK/ERK-AIRE and calcineurin/NFAT signalling axes: parallel mechanistic hypotheses

I hypothesise that the Ca²^+^ influx induced by CRET stimulation may activate the MAPK/ERK signalling cascade in mTECs. This hypothesis is informed by evidence that electromagnetic fields can activate ERK1/2 phosphorylation in non-thymic cell types, including keratinocytes and neurons (10, 11). However, I explicitly acknowledge that direct evidence for MAPK-mediated *AIRE* upregulation in mTECs has not yet been established.

The primary regulators of *AIRE* expression in mTECs are well-established to be the RANKL/RANK and CD40L/CD40 signalling axes, which activate NF-κB transcription factors (12—14). A highly conserved NF-κB-responsive enhancer element has been identified as critical for thymic *AIRE* expression (12). I therefore propose that CRET-induced Ca²^+^ signalling may converge on NF-κB activation via calcineurin/NFAT intermediaries as a co-equal and mechanistically distinct pathway to MAPK/ERK.

##### 2.1.1.1 The calcineurin/NFAT-NF-κB axis

Elevated intracellular Ca²^+^ activates calcineurin, a Ca²^+^/calmodulin-dependent phosphatase that dephosphorylates NFAT (Nuclear Factor of Activated T-cells) transcription factors, enabling their nuclear translocation (13, 14). In thymic epithelial cells, NFAT activation has been linked to the regulation of tolerance-associated gene programmes, and calcineurin signalling intersects with the NF-κB pathway at multiple nodes (15, 16). I propose that CRET-induced Ca²^+^ influx may activate this calcineurin/NFAT-NF-κB axis to upregulate *AIRE* expression, representing a mechanistic route that operates in parallel to, and independently of, the MAPK/ERK cascade. Both pathways will be systematically interrogated in the proposed NOD mouse studies (Section 4) using selective pharmacological inhibitors (FK506/cyclosporin A for calcineurin; PD98059/U0126 for MEK/ERK).

##### 2.1.1.2 Calcium-mediated apoptosis risk and mitigation

A critical safety consideration is that sustained or suprathreshold Ca²^+^ elevations can trigger mTEC apoptosis via calcineurin-dependent activation of pro-apoptotic pathways, including cytochrome c release and caspase-3 activation (17, 18). To mitigate this risk, the proposed CRET protocol employs a pulsed, rather than continuous, stimulation paradigm, with duty cycles designed to allow intracellular Ca²^+^ to return to baseline between pulses. Strict subthermal operation (ΔT < 1 °C) will be maintained throughout, as thermal co-stimulation potentiates Ca²^+^-mediated cell death. Dedicated safety monitoring endpoints — including mTEC viability assays (propidium iodide exclusion), TUNEL staining for DNA fragmentation, and caspase-3 activity measurement — will be incorporated into all preclinical experiments to ensure that the stimulation parameters selected for efficacy studies remain within the cytoprotective range (19, 20).

#### 2.1.2 Exposure parameters, dosimetric modelling and safety standards

The proposed 448 kHz CRET stimulation employs a dual-action waveform consisting of a 448 kHz sinusoidal carrier frequency modulated by a 10–40 Hz pulse envelope. The rationale for the 10–40 Hz modulation range is that it mimics endogenous biological signalling rhythms, such as neural oscillations and cellular calcium dynamics, and is hypothesized to enhance coupling with mTEC membrane properties (here used in a figurative, not physical sense), thereby optimising the bioelectronic effect. The wave shape is a pulsed sinusoidal wave, specifically optimized for deep tissue penetration to the anterior mediastinum while maintaining ultra-low energy deposition.

**Figure 1:**
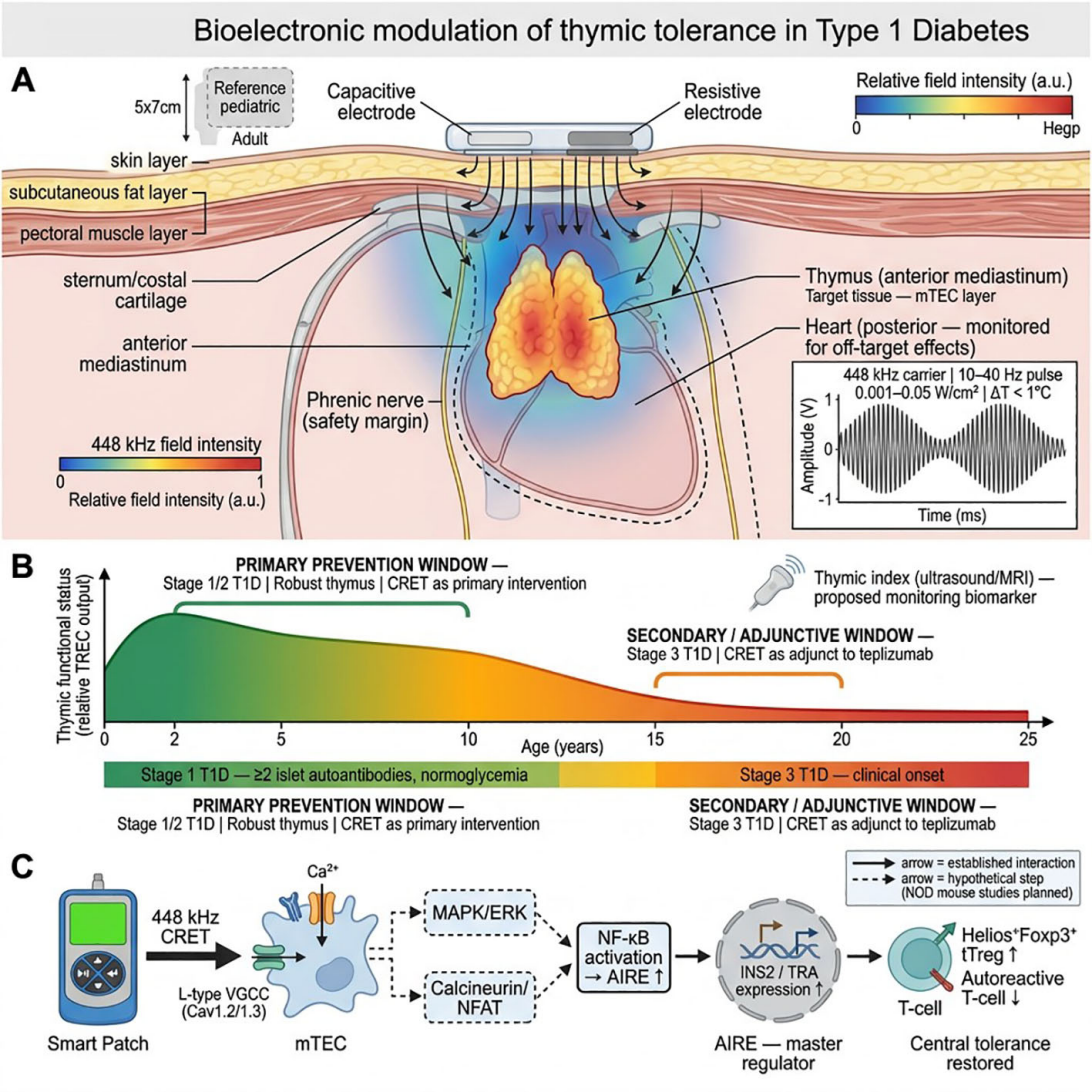
Bioelectronic modulation of the thymic “genetic mirror”: smart patch design, anatomical targeting, and therapeutic window staging. **(A)** Sagittal cross-section of the paediatric anterior thorax illustrating the anatomical relationship between the wearable 448 kHz CRET smart patch (applied at the manubrium sterni) and the target organ — the bilobed thymus in the anterior mediastinum. The colour gradient represents the simulated spatial distribution of the 448 kHz electromagnetic field (COMSOL Multiphysics; planned). Safety monitoring zones are indicated for the heart (middle mediastinum) and phrenic nerves (lateral pericardium), assessed against ICNIRP 2020 and IEEE C95.1 thresholds. Inset: dual-action waveform — 448 kHz sinusoidal carrier modulated by a 10–40 Hz pulse envelope; power density 0.001–0.05 W/cm² (athermal, ΔT < 1 °C). **(B)** Age-stratified therapeutic window framework. The curve represents the age-dependent decline in thymic functional output (TREC levels) from birth to adulthood. Two intervention windows are defined: the Primary Prevention Window (ages 2–10; Stage 1/2 T1D; robust thymic architecture; CRET as primary strategy) and the Secondary/Adjunctive Window (ages 10–18; Stage 3 T1D; accelerating involution; CRET as adjunct to teplizumab). Thymic index (ultrasound/MRI) is proposed as a monitoring biomarker. **(C)** Simplified cellular mechanism. Left-to-right flow from smart patch activation to central tolerance restoration. Solid arrows denote experimentally established interactions; dashed arrows denote hypothetical steps to be tested in the proposed NOD mouse studies. VGCC, voltage-gated calcium channel; mTEC, medullary thymic epithelial cell; NFAT, Nuclear Factor of Activated T-cells; TRA, tissue-restricted antigen; tTreg, thymus-derived regulatory T-cell.

From a safety perspective, the power density will be maintained at 0.001–0.05 W/cm², ensuring fully athermal operation with no thermal tissue effect. The intervention will be delivered via a wearable, battery-operated smart patch that provides continuous 24/7 steady-state delivery, allowing for sustained modulation of thymic function without requiring clinical visits or invasive procedures (**Figure 1**).

##### 2.1.2.1 Dosimetric modelling plan

Rigorous dosimetric modelling will be performed using COMSOL Multiphysics finite-element analysis, employing a validated anatomical phantom of the anterior chest wall and mediastinum derived from age-appropriate paediatric MRI data (21, 22). The model will characterise the spatial distribution of the 448 kHz electromagnetic field across the following tissue layers: skin, subcutaneous fat, pectoral muscle, sternum, anterior mediastinal adipose tissue and thymic parenchyma. Both capacitive electrode configurations (which preferentially deposit energy in superficial, high-impedance tissues) and resistive electrode configurations (which direct current through deeper, lower-impedance structures) will be modelled. The objective is to identify the optimal electrode geometry for maximising thymic field intensity while minimising energy deposition in adjacent structures (23, 24).

##### 2.1.2.2 Off-target safety assessment

Particular attention will be paid to two anatomically proximate structures: (i) the heart and pericardium, which lie immediately posterior to the thymus in the middle mediastinum, and (ii) the phrenic nerves, which course along the lateral pericardium. Simulated specific absorption rate (SAR) values at these structures will be compared against ICNIRP 2020 guidelines (general public reference levels: 2 W/kg averaged over 10 g tissue for the head and trunk) and IEEE Standard C95.1 thresholds (25, 26). Cardiac safety will also be assessed by monitoring electrocardiographic parameters (PR interval, QRS duration, QTc) in NOD mice during CRET stimulation sessions, using implantable telemetry (27, 28). All exposure parameters will be validated against these established safety standards before biological experiments commence.

### 2.2 Temporal dynamics of CRET-mediated thymic modulation

A critical distinction must be made between the acute, intermediate and chronic phases of CRET-mediated thymic modulation:

- Acute phase (seconds–minutes): CRET stimulation activates VGCCs, producing an immediate Ca²^+^ influx into mTECs. This triggers rapid phosphorylation events in MAPK/ERK and calcineurin pathways.
- Intermediate phase (hours–days): Sustained Ca²^+^ signalling leads to transcriptional activation of AIRE-dependent genes, including *Ins2*. This results in enhanced intrathymic insulin expression and presentation to developing T-cells, strengthening the negative selection checkpoint (29).
- Chronic phase (weeks–months): Repeated CRET sessions produce a sustained shift in thymic output: increased generation of insulin-specific Helios^+^Foxp3^+^ tTregs and reduced export of autoreactive effector T-cells. This structural remodelling of the peripheral immune repertoire constitutes the true therapeutic mechanism (30).

This temporal framework is essential for designing appropriate outcome measures in preclinical and clinical studies, and will guide the timeline of the proposed NOD mouse experiments.

### 2.3 Integrative pathway of the proposed mechanism

The proposed mechanism can be summarised as an integrative, multi-step pathway:

448 kHz RF stimulation → VGCC-mediated Ca²^+^ influx → activation of MAPK/ERK and calcineurin/NFAT signalling cascades → NF-κB activation → upregulation of *AIRE* → increased *Ins2* gene expression → enhanced insulin peptide presentation → induction of insulin-specific Helios^+^Foxp3^+^ regulatory T cells (Tregs) and reinforcement of central immune tolerance.

**Figure 2:**
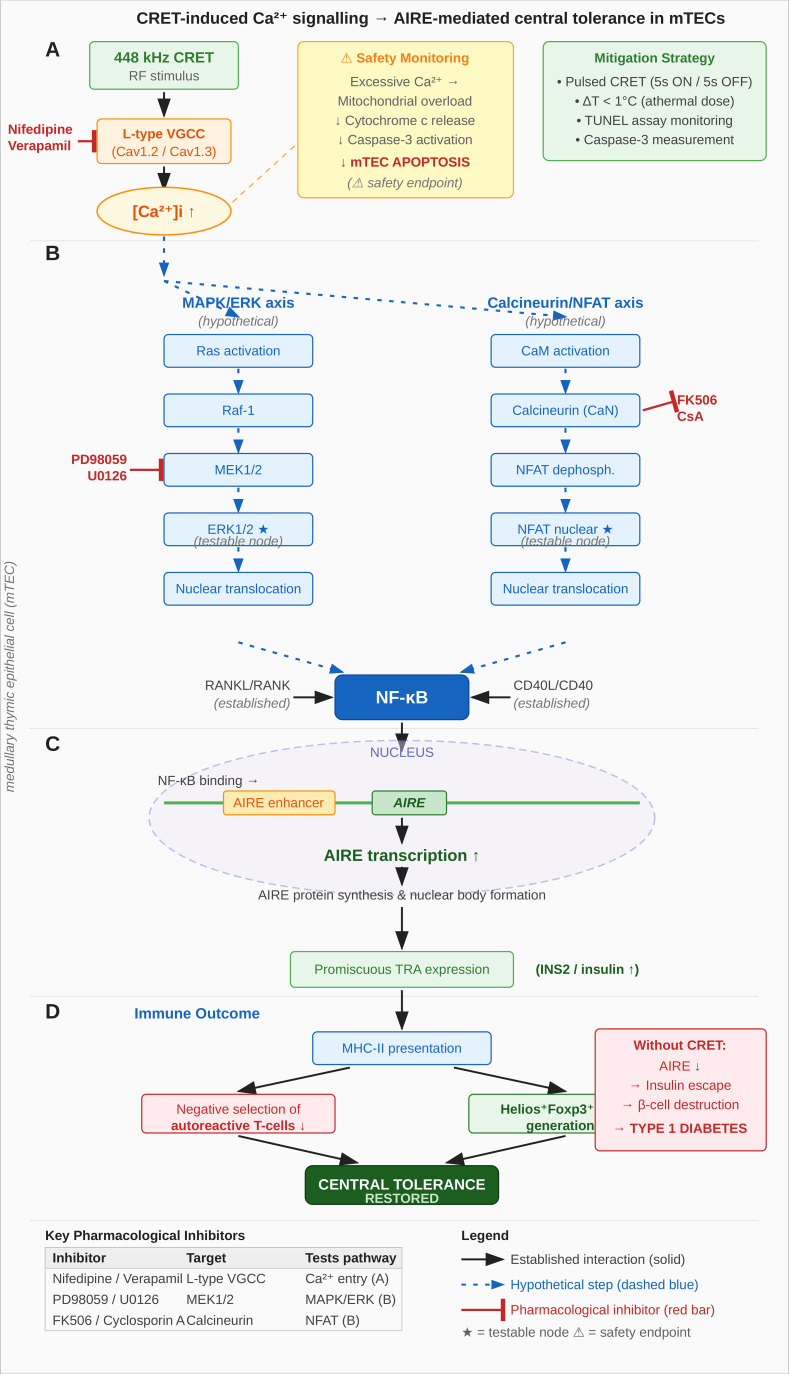
Full molecular cascade linking CRET-induced Ca²^+^ signalling to AIRE-mediated central tolerance in medullary thymic epithelial cells (mTECs). **(A)** Stimulus and Ca²^+^ entry. Subthermal 448 kHz CRET fields (ΔT < 1 °C) are hypothesised to activate L-type voltage-gated calcium channels (Cav1.2/Cav1.3) on the mTEC membrane, producing localised intracellular Ca²^+^ elevation ([Ca²^+^] ↑). Pharmacological blockade with Nifedipine or Verapamil will confirm channel involvement. A safety branch illustrates the apoptotic risk of sustained Ca²^+^ overload (cytochrome c release → caspase-3 activation); this is mitigated by pulsed stimulation, strict athermal operation and TUNEL/caspase-3 monitoring. **(B)** Parallel signalling pathways (hypothetical). Elevated (Ca²^+^) is proposed to activate two co-equal, mechanistically distinct cascades: pathway A — the MAPK/ERK axis (Ras → Raf-1 → MEK1/2 → ERK1/2 nuclear translocation; inhibited by PD98059/U0126) and pathway B — the Calcineurin/NFAT axis (calmodulin → calcineurin → NFAT dephosphorylation → nuclear translocation; inhibited by FK506/cyclosporin A). Both pathways converge on NF-κB activation. **(C)** Established NF-κB–AIRE axis. NF-κB activation — also driven by the well-characterised RANKL/RANK and CD40L/CD40 signalling inputs — drives binding to the conserved NF-κB-responsive *AIRE* enhancer, upregulating *AIRE* transcription and protein synthesis. AIRE-dependent promiscuous gene expression increases intrathymic insulin (*INS2*) and other tissue-restricted antigen (TRA) levels. **(D)** Immune outcome. INS2/TRA peptides presented on MHC-II molecules reinforce negative selection of autoreactive T-cells and promote the generation of stable Helios^+^Foxp3^+^ thymus-derived regulatory T-cells (tTregs), collectively restoring central immune tolerance. In the absence of CRET intervention, reduced *AIRE* expression permits insulin-specific T-cell escape and progressive β-cell destruction, culminating in T1DM. Arrow conventions: Solid arrows (→) denote experimentally established interactions; dashed blue arrows (⇢) denote hypothetical steps to be interrogated in the proposed NOD mouse studies (Section 4). Red inhibitory bars (⊣) indicate pharmacological blockade points. ★ marks key, independently measurable testable nodes. ⚠ marks safety monitoring endpoints. CaM - calmodulin; CaN - calcineurin; CRET - Capacitive-Resistive Electric Transfer; mTEC - medullary thymic epithelial cell; NFAT - Nuclear Factor of Activated T-cells; TRA - tissue-restricted antigen; tTreg - thymus-derived regulatory T-cell; VGCC - voltage-gated calcium channel.

Several steps in this pathway are well-supported by existing literature. NF-κB is a well-established regulator of *AIRE* expression in mTECs, and AIRE is known to drive promiscuous expression of tissue-restricted antigens, including insulin. The importance of intrathymic insulin expression for the development of insulin-specific Tregs has been demonstrated in both mouse models and human genetic studies. However, other steps remain speculative and require experimental validation — specifically, the direct effect of CRET-induced Ca²^+^ signalling on MAPK/ERK and calcineurin pathways in medullary thymic epithelial cells. These hypothetical steps are indicated in **Figure 2** using dashed arrows, whereas solid arrows represent experimentally supported interactions.

## 3 Contextualizing CRET within the immunotherapy landscape

The 448 kHz CRET approach and CAR-Treg cell therapies share a fundamental therapeutic goal: the restoration of antigen-specific immune tolerance to insulin. However, this comparison should be understood as a conceptual analogy rather than a claim of direct equivalence or superiority.

CAR-Tregs achieve exquisite cellular-level antigen specificity through engineered T-cell receptors that directly recognise insulin peptide-MHC complexes (31). This represents a precision that the CRET approach, operating at the tissue level, cannot claim to match at the single-cell level. The distinct advantages of the CRET approach are:

- Non-invasiveness and paediatric suitability: no cell extraction, manufacturing or infusion or infusion required
- Continuous, adjustable modulation: wearable patch allows titrated, ongoing stimulation
- Thymic re-education: acts upstream at the source of tolerance induction, not downstream
- Cost and accessibility: potentially far more scalable than individualised cell therapies

### 3.1 Epigenetic stability of tTregs versus CAR-Tregs

A critical and underappreciated advantage of thymus-derived Tregs, generated through CRET-mediated thymic re-education, is their superior epigenetic stability compared to *ex vivo* engineered CAR-Tregs. The lineage stability of tTregs is anchored by stable CpG demethylation of the *FOXP3* conserved non-coding sequence 2 (CNS2), also termed the Treg-specific demethylated region (TSDR) (32, 33). This epigenetic mark is established during thymic development under the influence of the thymic microenvironment and is maintained throughout the lifespan of the cell, ensuring sustained *FOXP3* expression and suppressive function even under inflammatory conditions (34).

In contrast, CAR-Tregs generated by *ex vivo* retroviral or lentiviral transduction of peripheral CD4^+^ T-cells frequently retain partial CNS2 methylation, resulting in *FOXP3* expression that is dependent on the transgenic promoter rather than the endogenous epigenetic programme (35, 36). Under pro-inflammatory conditions encountered in the T1DM lesion, this epigenetic instability can lead to *FOXP3* downregulation and functional conversion of CAR-Tregs into pro-inflammatory effector cells — a phenomenon termed “Treg plasticity” (37). Supraphysiological *FOXP3* overexpression strategies have been proposed to partially mitigate this instability in CAR-Treg products, but the long-term durability of this approach *in vivo* remains to be established (38, 39).

The Helios^+^ phenotype provides an additional marker of tTreg identity and stability. Helios, an Ikaros family transcription factor, is expressed at high levels in thymus-derived Tregs and is associated with enhanced suppressive capacity, superior peripheral fitness, and resistance to inflammatory reprogramming (43—46). CRET-induced thymic re-education is specifically designed to promote the generation of Helios^+^Foxp3^+^ tTregs — cells that carry both the epigenetic stability of CNS2 demethylation and the functional robustness conferred by Helios expression. This dual epigenetic and phenotypic stability represents a key mechanistic advantage of the thymic re-education strategy over approaches that rely on *ex vivo* Treg expansion or engineering.

I acknowledge that rigorous dosimetric modelling and RF field distribution validation will be essential to characterise the actual spatial precision of CRET stimulation within the thymic tissue. This is addressed in the experimental design presented in Section 4.

## 4 Proposed experimental validation: nod mouse studies

To test this hypothesis, a series of experiments using the NOD (Non-Obese Diabetic) mouse model is proposed. The NOD mouse spontaneously develops Type 1 Diabetes and is the gold standard for preclinical T1DM research.

### 4.1 Safety and field characterisation

Before any biological experiments begin, the 448 kHz CRET device will be confirmed to operate safely within the target tissue. This includes measuring the actual distribution of the electromagnetic field within the thymus, verifying that tissue temperature does not rise by more than 1 °C during stimulation (subthermal operation), and examining thymic tissue under the microscope after treatment to confirm no structural damage has occurred.

### 4.2 Mechanistic studies

The effect of CRET stimulation on signalling pathways inside thymic epithelial cells will be investigated. Specifically, the stimulation will be tested for activation of the MAPK/ERK pathway and the calcineurin/NFAT pathway, and whether either leads to increased AIRE protein activity and, consequently, greater insulin expression within the thymus.

Selective pharmacological inhibitors (FK506/cyclosporin A for calcineurin; PD98059/U0126 for MEK/ERK) will be used to confirm which signalling pathway is the primary mediator.

### 4.3 Immune and therapeutic outcomes

The key question is whether CRET treatment leads to a measurable improvement in immune tolerance. Treated NOD mice will be monitored over 16 weeks, tracking:

- Regulatory T-cell levels: whether protective Helios^+^Foxp3^+^ Tregs increase in the thymus and peripheral lymph nodes
- Diabetes onset: whether treated mice develop T1DM later or less frequently than untreated controls (Kaplan-Meier analysis, *n* = 20 per group)
- Pancreatic inflammation: whether the degree of immune cell infiltration into the pancreas (insulitis) is reduced at study endpoint

Together, these three stages will establish not only that CRET is safe, but also elucidate the mechanism by which it works and demonstrate a measurable therapeutic benefit in a well-validated preclinical model.

## 5 Discussion

The proposed bioelectronic modulation of thymic function represents a novel therapeutic paradigm. While the emerging field of bioelectronic medicine has primarily advanced through neural circuit-based immunomodulation via vagus nerve stimulation (40), the present approach extends this paradigm towards direct, non-neural cellular modulation of thymic epithelial cells. Unlike current T1DM management strategies that address glycaemic control or peripheral immune suppression, the 448 kHz CRET approach targets the fundamental immunological defect: insufficient central tolerance to insulin.

The shared embryonic origin of the thymus and pancreas from the primitive foregut endoderm provides a biological rationale for why the thymus retains the capacity to express insulin and why this expression is critical for preventing T1DM (1, 2). The AIRE protein is the master regulator of this promiscuous gene expression programme (41), and its activity is governed by well-characterised signals from RANK^+^ lymphoid tissue inducer cells and CD40L^+^ thymocytes (42, 43).

This temporal framework is critical for informing the design of future preclinical and clinical studies. The therapeutic benefit of repeated CRET sessions is expected to accrue over weeks to months, as the chronic phase of thymic re-education produces a measurable shift in the peripheral immune repertoire. This is analogous to the delayed but durable effects seen with teplizumab (anti-CD3) therapy, where a short course of treatment produces years of benefit through sustained changes in T-cell subset composition (44).

I acknowledge the significant mechanistic uncertainties that remain. The MAPK/ERK-AIRE axis is presented as a testable hypothesis, not an established mechanism. The experimental design outlined in Section 4 is specifically structured to test and potentially falsify this hypothesis. If MAPK/ERK inhibition does not reduce CRET-induced *AIRE* upregulation, this would indicate that alternative pathways — most likely the calcineurin/NFAT-NF-κB axis — are the primary mediators, and the hypothesis would be revised accordingly.

An important consideration for the translational potential of this hypothesis is the substantial physiological variability in thymic responsiveness across species, developmental stages, and individual genetic backgrounds. While the NOD mouse model shares many immunological features with human Type 1 Diabetes, there are important differences in thymic architecture, mTEC gene expression profiles and the kinetics of autoimmune progression between mice and humans. These species-specific differences mean that findings in NOD mice will need to be carefully validated in human-relevant experimental systems before clinical translation can be pursued.

### 5.1 Therapeutic window: a rigorous age-stratified framework

Age-related thymic involution represents one of the most significant biological challenges for this intervention. I provide a rigorous, age-stratified framework that explicitly delineates two distinct therapeutic windows based on human thymic involution kinetics and T1D disease staging:

- Primary prevention window — Stage 1/2 T1D (ages 2–10 years): this window targets pre-symptomatic, islet autoantibody-positive children who exhibit Stage 1 (≥ 2 islet autoantibodies, normoglycaemia) or Stage 2 disease (dysglycaemia, preserved beta-cell mass > 50%) (45, 46). At this developmental stage the thymus retains robust architecture with high mTEC density, preserved *AIRE* expression, and maximal capacity for central tolerance induction (47, 48). Human thymic output, quantified by T-cell receptor excision circles (TRECs) in peripheral blood, remains high during early childhood and declines progressively after age 10, with a 50% reduction in thymic mass documented by age 15 (49). This window offers the greatest potential for reshaping the autoreactive T-cell repertoire and preventing progression to clinical diabetes, and represents the primary target population for this intervention.
- Secondary/adjunctive window — Stage 3 T1D (adolescence/young adulthood): this window applies to adolescents and young adults at clinical onset (Stage 3: symptomatic hyperglycaemia, significant β-cell loss). By adolescence, thymic involution accelerates markedly, with documented reductions in thymic mass, mTEC populations and tissue-restricted antigen expression (50, 51). Central tolerance capacity is substantially diminished, limiting the efficacy of thymic modulation as a monotherapy. In this scenario, CRET would serve as an adjunctive strategy alongside emerging disease-modifying therapies to preserve residual β-cell function rather than as a primary tolerance-induction strategy (52–56).

Individual genetic backgrounds may also influence thymic responsiveness. Polymorphisms in the *INS* gene (particularly the VNTR region), HLA haplotypes and other immune-related loci are known to affect intrathymic insulin expression and T1DM susceptibility, and should be considered as stratification variables in future studies. The design of future clinical trials should incorporate age-stratified endpoints and thymic imaging biomarkers - such as thymic index measured by ultrasound or MRI - to objectively assess thymic function and treatment response, and to identify individuals most likely to benefit from this intervention.

## 6 Conclusion

The 448 kHz CRET approach to thymic modulation represents a hypothesis-driven, mechanistically grounded, and experimentally testable strategy for disease-modifying intervention in T1DM. By targeting the root cause of autoimmunity — the failure of central tolerance — rather than its downstream consequences, this approach offers a fundamentally different therapeutic logic.

Central to this framework is the proposed VGCC→Ca²^+^→MAPK/ERK and calcineurin/NFAT→NF-κB→*AIRE* signalling cascade, through which subthermal radiofrequency fields may amplify intrathymic insulin expression and reinforce negative selection. This cascade is presented as a falsifiable model: each node — from calcium influx kinetics to AIRE-driven *INS* gene transcription and Helios^+^Foxp3^+^ Treg differentiation — constitutes an independently measurable endpoint. The NOD mouse model, with its well-characterised autoimmune phenotype and thymic pathology, provides an ideal preclinical platform for systematic validation of this pathway.

If preclinical validation is achieved, the 448 kHz smart patch offers a favourable translational profile for paediatric endocrinology: non-invasive, continuously tunable and compatible with existing insulin therapy. Its potential to preserve residual β-cell mass during the critical window of disease onset — particularly relevant in children diagnosed at an early age within the primary prevention window (Stage 1/2, ages 2–10) — positions it as a candidate for future early-intervention trials. Nonetheless, the translational pathway from hypothesis to clinical application requires rigorous dose-response characterisation, long-term safety assessment and mechanistic confirmation before any human application can be considered. This work is offered as a structured scientific foundation for that investigative programme.

## Data Availability

The original contributions presented in the study are included in the article/**Supplementary Material**. Further inquiries can be directed to the corresponding author.
