# Supplementary figures and images for: Bioelectronic modulation of the thymic “genetic mirror”: 448 kHz radiofrequency stimulation as a novel strategy for immune tolerance induction in type 1 diabetes
Source: Front Immunol. 2026 Jun 15;17:1851942. doi: 10.3389/fimmu.2026.1851942 (PMC13310697; doi:10.3389/fimmu.2026.1851942)

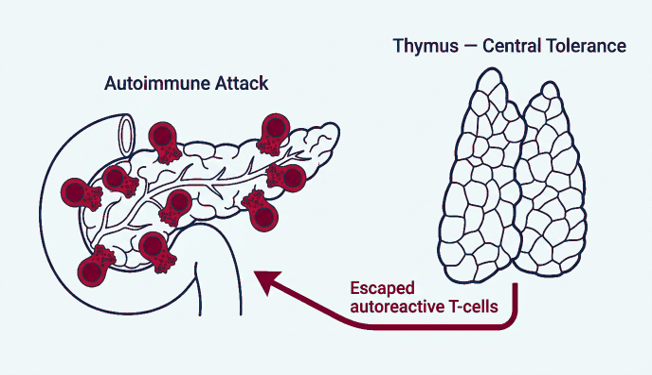

Supplement: Supplementary file 1 [file Image1.png]

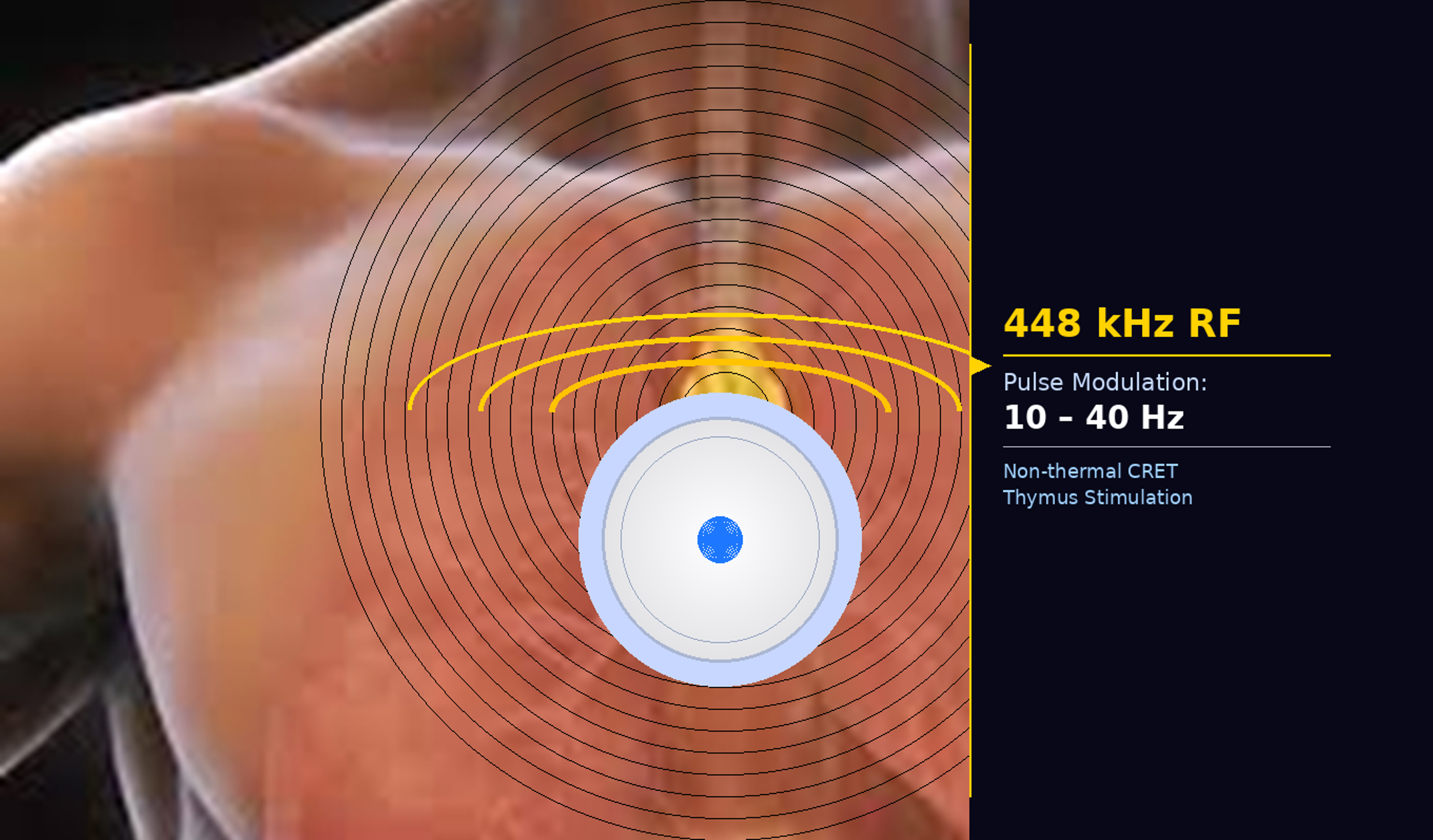

Supplement: Supplementary file 2 [file Image2.png]
